# The Highest Branch of Medicine

**Published:** 1869-10

**Authors:** 


THE HIGHEST BRANCH OF MEDICINE.—It is a curious reflection that, in theory, the aim and objective point of the three learned professions is to do away with the necessity of their existence. The priest or preacher seeks to reform the world, to make it so righteous that there will be no further need of admonition and exhortation; the lawyer is constantly striving to make crime so unpleasant, and to popularize justice so thoroughly, that the statute book and the jail will no longer be required; the physician recognizes the prevention of disease as the highest purpose of his calling. If he could succeed, there would be little or nothing left for him to do; for prevention would ask little beyond individual knowledge.

Such being the most elevated object of medical science, it is worth inquiry how best it may be attained. Year by year the belief in specifics, or in any doctrine of signatures, has been diminishing, until now we may consider it extinct. We trust in the natural powers more, in the individual capacity more, less and less in foreign impulses communicated to the system. This is true in prophylaxis as well as treatment. Thorough, careful, systematic hygienic remedies are better than any drug or preventive agent.

It is one of the best signs of the times that the care of the body in health has been attracting the attention of medical men of late years, as much as the care of it in disease. Personal hygiene should occupy quite as prominent a place in medical discussions as public hygiene. For every valid effort in this as in political life, must start from the individual.

We should, therefore, as a body, encourage the dissemination of correct views on anatomy and physiology, we should strive to introduce such studies into schools, and we should give more attention to the education of adults in these matters. Domestic medicine is not our admiration. Everybody his own doctor is a calamitous proposition. Certain simple and harmless remedies it were well to have known widely, and procedures in cases of sudden accident. Beyond this the public need not be instructed.

But they should know as much as they will learn about the structure of the body, and how to preserve it in the very best condition.

